# Water, Protons, and the Gating of Voltage-Gated Potassium Channels

**DOI:** 10.3390/membranes14020037

**Published:** 2024-01-29

**Authors:** Alisher M. Kariev, Michael E. Green

**Affiliations:** Department of Chemistry and Biochemistry, The City College of New York, New York, NY 10031, USA; akariev@ccny.cuny.edu

**Keywords:** water, protons, ion channel voltage gating, proton wire, ion hydration

## Abstract

Ion channels are ubiquitous throughout all forms of life. Potassium channels are even found in viruses. Every cell must communicate with its surroundings, so all cells have them, and excitable cells, in particular, especially nerve cells, depend on the behavior of these channels. Every channel must be open at the appropriate time, and only then, so that each channel opens in response to the stimulus that tells that channel to open. One set of channels, including those in nerve cells, responds to voltage. There is a standard model for the gating of these channels that has a section of the protein moving in response to the voltage. However, there is evidence that protons are moving, rather than protein. Water is critical as part of the gating process, although it is hard to see how this works in the standard model. Here, we review the extensive evidence of the importance of the role of water and protons in gating these channels. Our principal example, but by no means the only example, will be the K_v_1.2 channel. Evidence comes from the effects of D_2_O, from mutations in the voltage sensing domain, as well as in the linker between that domain and the gate, and at the gate itself. There is additional evidence from computations, especially quantum calculations. Structural evidence comes from X-ray studies. The hydration of ions is critical in the transfer of ions in constricted spaces, such as the gate region and the pore of a channel; we will see how the structure of the hydrated ion fits with the structure of the channel. In addition, there is macroscopic evidence from osmotic experiments and streaming current measurements. The combined evidence is discussed in the context of a model that emphasizes the role of protons and water in gating these channels.

## 1. Introduction

Massive effort has gone into understanding the role of water, and of protons, in the function of a variety of proteins and biological surfaces. This has been reviewed extensively [1,2], with multiple specific applications in many forms of proteins, membranes, and other types of biological surfaces. In particular, ion channels, which, along with transporters, are responsible for communication between the cell and its surroundings, as well as certain cell functions, such as the nerve impulse, have been the focus of massive effort. The same is true of water structure and proton transport. The fundamental idea of the motion of protons through water has been known for over two centuries; Grotthuss, even before it was known that water was H_2_O (he thought it was HO), had the basic idea, published in 1806, and the transit of protons through water by exchange with neighboring molecules is still known as the Grotthuss mechanism. New information on hydrogen bonding, and the role of the amino acid side chains of protein as a section of the hydrogen bond chain that constitutes a water wire in biological systems, has come along at an increasing pace. This includes new information on ion channels. Grotthuss could have had no idea of any of this, and he obviously could not have imagined ion channels, but now we can put the parts together to form a coherent picture of the role of proton transport at protein surfaces and interior pores. Together with the structural work on proteins of the past half century, and especially the last quarter century for membrane proteins, we are in a position to apply our knowledge of water structure and proton transport to ion channels.

Channels for ions other than H^+^ generally have an aqueous pore through which ions, or water molecules themselves, can travel when the pore is open. In order to be functional, there must be a way to open the channel for conduction and close the channel when it should not be conducting ions. The mechanism of this step, called gating, is central to channel function, and much effort has gone into working out the details of the mechanisms for the various types of channels that exist; even H^+^ conducting channels must have a gating mechanism, although they lack an aqueous pore that could transmit anything bigger than H^+^. Studies on channel gating and conduction have a long history [3], but much progress has been made in the past quarter century, starting with determination of the structure of a bacterial potassium channel by MacKinnon and coworkers in 1998 [4]. By now, the structure of many channels has been determined by X-ray crystallography, or some other techniques, including cryo-electron microscopy and, for smaller channels, NMR. Voltage-gated channels have presented a particular challenge in that neither X-ray crystallography nor cryo-EM is directly usable with the application of voltage. Thus, almost all voltage-gated channels have a known structure in the open state (zero voltage), but the closed state, which requires the application of a voltage, has not been determined experimentally. In a couple of channels, it may be that the closed state exists at zero potential, but these are hard to compare to the usual channels. There have been a huge number of computational attempts, mostly using molecular dynamics; we have, in the past, raised doubts about how these are to be interpreted [5]. In addition to the channels that allow ions to cross the membrane, there are channels that are specialized to allow only water [6], or only protons [7,8], to cross.

**Figure 1:**
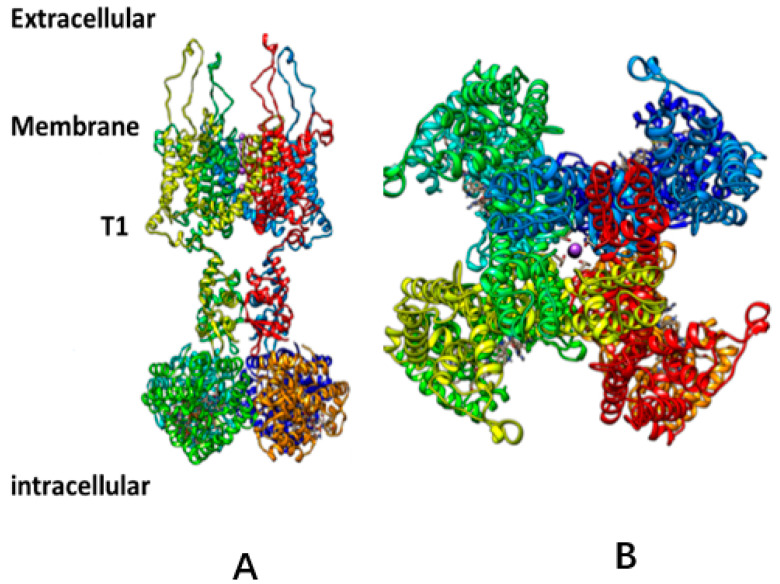
General view of a Kv1.2 channel from the 3Lut X-ray coordinates [10]. (**A**): View from the membrane (side view). Two of four domains are shown, with front and back domains deleted to allow the structure to be seen. (**B**): A 90° rotation of the channel, showing the pore. In neither (**A**) nor (**B**) are individual amino acids shown. Each domain is a different color. The T1 intracellular section is known to be important in gating; this section is visible in (**A**). Neither (**A**) nor (**B**) make it clear that the linker is not entirely continuous unless the water is included; water is not shown in these figures. The large intracellular section shown in (**A**) is not known to be related to gating in either the standard model or the model proposed here. The ion can be seen in the center of the pore in B, and its four-coordination is visible. The section labels are for (**A**).

A consensus on gating the voltage-gated channels, based on several assumptions concerning the nature of the voltage-sensing domain, has developed, in which a segment of protein crosses the membrane to go from open to closed states [9]. Figure 1 shows the general structure of a channel with the consensus-determined gating mechanism. The assumptions for this model are based on a seemingly straightforward interpretation of a number of experiments, but these interpretations actually can be interpreted in more than one way; in other words, the assumptions that are built into the standard interpretation require reconsideration [5].

Not only is gating fundamental for the understanding of channel function, but it is a possible target for drugs that might regulate channels and thus ion concentrations in cells. Here, we are particularly interested in channels that transport potassium ions, with the gating occurring in response to voltage changes; however, there are many hundreds of types of channels, some of which gate in response to ligands, mechanical stress, temperature changes, or some combination (for example, TRPV1 both senses temperature and a ligand, capsaicin, the hot pepper ligand [11]). The opening of the voltage-gated channels responsible for the nerve impulse is preceded by a “gating current” of about ten to thirteen charges that move up (where “up” means move in the extracellular direction, and “down” means to move in the intracellular direction—we shall adopt this convention throughout this paper) when the transmembrane voltage is removed [12,13,14,15] and down when a voltage is applied to close the channel. The channel itself, both for potassium and sodium channels, has four-fold symmetry, exact in the case of the potassium channel and approximate for the sodium channels. There are four voltage-sensing domains (VSD), each with four transmembrane protein segments and a central pore composed of two transmembrane segments from each of the four domains. One transmembrane segment in each VSD contains several positively charged amino acids, and it has long been believed that the opening and closing of these channels depended on a physical response of the positively charged transmembrane segment, which is taken to move “up” to open, or “down” to close [16,17,18], the gate of the channel. The structures of the potassium channels have been found only in the open state. The X-ray structures cannot be obtained with an applied voltage, so the structure of the resting or closed state remains to be determined as of the time of this writing, although attempts have been made, and are being made, to determine this using other techniques. So far, all attempts with a K_v_ channel have been unsuccessful, but it is reasonable to expect that, at some point, it will succeed and that the question of the gating mechanism will be largely settled. Wallace and coworkers have studied prokaryotic sodium channels, which appear to be closely related to eukaryotic sodium channels. Sodium channels are analogous to potassium channels, although they are not exact tetramers, as potassium channels are. Wallace and coworkers found that hinge motions of the pore helix S6 and the C-terminal domain were involved with gating and affected gate diameter, but there was essentially no S4 movement between open and closed structures [19,20]. With the absence of S4 motion in this channel, the gating current cannot be accounted for by simply moving the S4 arginines. In the standard models, these provide the positive charge which is supposed to provide the transient capacitive current (gating current) that precedes channel opening. The gating current has been measured and the magnitude of the charge that moves determined for the channels we are discussing [12,13].

Without moving arginines, how is this possible? The gating current is a measured phenomenon, so positive charges must move. S4 motion has become essentially canonical, at least in a generic sense; however, this class of gating models, while generally accepted, has not been directly confirmed by any structural evidence, and it is possible to interpret the evidence as the gating current being a consequence of proton motion. The S4 mechanism was first suggested over a quarter century ago; the motion is supposed to be transmitted to the somewhat distant (>10 Ả) gate through an intracellular linker, with a down motion pushing on the protein to mechanically close the gate and an up motion pulling the gate open. The details of how this is accomplished have never been worked out, and many complicating experimental results are difficult to explain via this type of mechanism. Several disparate versions, based on different experiments, have been proposed, and the early work, which led to essentially all the present versions, has been very nicely summarized by Tombola et al. [21]. It remains to be shown that the evidence that led to the original mechanical model can be explained by the proposed proton model as well. Significant new evidence specifically supporting the mechanical model has not appeared since, although practically all of the hundreds of experiments reported since tend to be interpreted in terms of this model.

Kariev and Green, in a series of papers [5,22,23,24], considered the possibility that the voltage gating of ion channels depends on *protons* moving under the influence of the electric field to generate the measured gating current, with the transmembrane segment stationary as the field changes—in this model, protons move, while the protein backbone remains essentially static, although the rotation of side chains is necessary to help transmit the protons. We have supplied principally quantum calculations in support of the proton gating model, together with consistent interpretations of the experiments that have been proven to be difficult to explain on the standard models. We have also discussed the experiments that are the basis of the standard model, pointing out that alternate explanations are possible for all such results [5]; none of the experiments on which the standard model is based offers proof that is definitive. The evidence for the importance of protons in gating, and with these, water, both as part of a transport chain for the protons and as part of the structure of the gate itself, is worth a full discussion, and here, we bring together a number of experiments and calculations that show that water is important in both respects and that this is probably because of its contribution to the transit of protons.

There is remarkable conservation of one section of the potassium channel, the selectivity filter (SF), which is distant from the gate; the SF empties into the extracellular space, while the gate is at the intracellular surface. The SF sequence appears to be not only the same in a bacterial channel [4] and eukaryotic channels, but even in viral channels [25,26]. The strong conservation of this channel section suggests the fundamental importance of the structure and also of this general type of channel. However, the relation of this section to the gate appears to be remote. Inactivation, however, shuts down conduction, and that does appear to be related to the SF. With this being noted, we return to the gate, and with it, the VSD and linker.

Specific evidence pertaining to the roles of water and protons in these channels comes from H_v_1, a proton channel analogous to the VSD, from mutational experiments on both the channels of direct interest and, in some instances, of comparable channels, from the use of D_2_O, from pH adjustments, and even from some macroscopic experiments involving a streaming current and osmotic pressure. To the extent that these can be considered separately, we will try to organize them so as to allow the consideration of the different effects. Not surprisingly, experiments that concern the formation of water networks and hydrogen bond networks and specific forms of protonated water species are particularly important, along with the hydration of the sodium and potassium ions, especially in confined spaces, such as the interior of channels. Ana-Nicoleta Bondar has given a more general review of allosteric effects related to proton transfer with a hydrogen bond network [27].

## 2. Evidence Pertaining to a Possible Role for Water and Protons in Gating Channels, Especially the Voltage-Gated Potassium Channels Shaker and K_V_1.2

*(1)*  *The H_v_1 channel as an analogue of the VSD:* Another major consideration is that some types of channels are known to conduct protons. The best known example is the H_v_1 channel, which has a structure that is, in many ways, similar to the VSD [28,29,30,31]; however, there exist significant differences (for one thing, H_v_1 is a dimer, and the proton path would differ somewhat from that in the VSD of the mammalian K_v_1 channel; however, the fundamental structure is similar, and the upper part of the channel strongly resembles that of the VSD of the K_v_1.2 channel). Although it is not generally considered that this challenges the standard model, it is difficult to see why it does not; it provides a similar path that could be taken by protons [32]. H_v_1 is the subject of multiple studies, and there is still no general agreement on the gating mechanism [29,33,34,35,36,37,38]. It appears that protons contribute to the gating current [39]. The wide variety of suggested conformations implies that there is a complex set of states or that the gating mechanism involves something other than a conformational transformation of the channel. A number of residues, when mutated, change the gating, transport, and other properties of the channel [28,29,40,41]. While the H_v_1 channel is not identical with the VSD of a K_v_ channel, the general structure is similar enough that it is reasonable to conclude that the VSD of a K_v_ channel can conduct protons in response to an electric field. In other words, this structure strongly suggests that the VSD has proton transport as a core function. The H_v_1 channel appears to have an upper section that is very similar to the VSD. In our earlier calculations, we found that the second arginine could be a source of protons from an arg-glu-tyr (REY) triplet of amino acids [42]. This is also where most of the electric field drops [43], reaching values close to 10^8^ V m^−1^, so that there is almost no field across the remainder of the VSD (10^8^ V m^−1^ × 7 Ả = 70 mV, leaving nothing for the rest of the VSD). At a field of 10^8^ V m^−1^, non-linear effects on ionization become important [44] (thus, one must be very careful in evaluating molecular dynamics results that use such a high field for the entire VSD). Our calculations therefore accord with the known effects. The H_v_1 channel path diverges from that of the VSD below this point, as the path of the proton would be different in the two cases, but the analogy of the H_v_1 still strongly suggests that the upper section should be able to produce a proton and that the proton should push additional protons to the inward side of the membrane. The H_v_1 channel voltage sensor is also regulated in part by pH [45], which makes sense if the proton current starts with a proton transfer. It must also be possible to close the channel, and this is usually attributed to a hydrophobic gasket [40]; mutations in this gasket, to less hydrophobic amino acids, allows some of the proton current through. The VSD to allow just three protons through in some tens of microseconds could easily be possible with a very limited driving force. Electrostatic control of the proton current is consistent with pH control in that the field is altered by the state of protonation [46]. MD simulations suggest that a water wire exists in H_v_1 but not in the VSD of a voltage-gated channel [47]. Modeling based on a putative (and probably correct) closed state of H_v_1 suggests the existence of a water wire in the open state but a hydrophobic plug in the closed state [48,49]. Much of the electrophysiology of H_v_1 has been worked out by Thomas DeCoursey and colleagues. Although DeCoursey assumes protein motion in gating, his data are consistent with pH and electrostatic control that does not require motion [35]. While we cannot go through all the details of the gating and conduction of the H_v_1 channel, there is also strong evidence for the importance of water as a bridge between energy minima for the proton [50] and, more interestingly, for fluctuations of water as a factor in proton transport [36]. At this point, there has not been a test of the effects of fluctuations with the presence of a voltage. Early—pre 1980s—noise measurements notwithstanding, the interpretation of these results is not transparent.

The H_v_1 results can be summarized as follows: (i) pH and voltage combine to control gating. (ii) In the open state, there is a water wire, or a proton wire that is partly water and partly a protein side chain, or fluctuations in water that allow a complete water wire. (iii) In the closed state, there appears to be a hydrophobic plug to block the proton current; the efficiency of this plug, including the effect of fluctuations in water density, in voltage, or of a proton leak, has not been determined. This may be relevant to the VSD of a voltage-gated potassium channel in that the VSD should be able to transmit protons and water should penetrate from either end. The existence of a hydrophobic plug may be analogous to what happens with a VSD, but the leak required to allow a proton current is small enough to be consistent with a model in which protons are the gating current.

**Figure 2:**
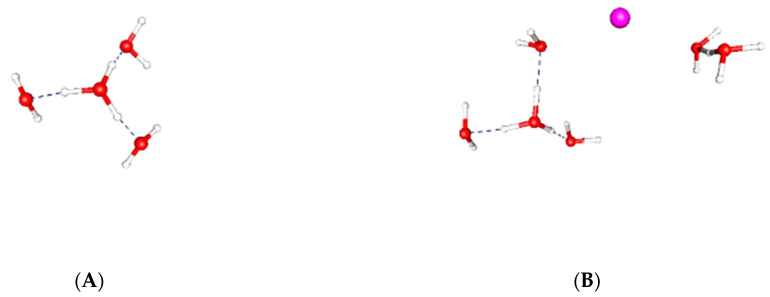
(**A**) An Eigen ion, H_9_O_4_^+^, (**B**) A K^+^ with an Eigen ion plus a Zundel ion on the right in a configuration taken from an optimization.

*(2)*  *Other channels, not so closely related to the K_v_1 VSD, also transport protons*. These include bacteriorhodopsin [51], cytochrome c [52,53,54], and the M2 channel of the influenza virus [55,56,57,58]. Bacteriorhodopsin has water as a critical component in the proton path [59,60,61,62,63]. So does cytochrome c [64,65,66,67,68,69,70,71,72,73] and the M2 channel of the influenza virus [56,74,75,76,77,78]. Much of the work on the influenza M2 channel concerns a water network; water networks include hydrogen bonding that involves extensions of hydronium ions (Eigen ions, Zundel ions [79,80,81,82]). Enough channels are known to transport protons to make it clear that proton transport in channels is fairly common and can be part of water networks. Again, the arrangement of side chains is part of the transport mechanism, but in none of these is a significant rearrangement of the backbone of the protein required. We have completed a set of nine optimizations of the gate region of the channel and found that Eigen and Zundel ions both appear when the ion approaches the PVPV level of the gate. Figure 2A illustrates an Eigen ion, while Figure 2B shows a potassium ion with one Eigen and one Zundel ion in a configuration that actually formed in an optimization.
*(3)*  *Proton wires, coupled to water networks and proteins:* All of the channels mentioned in Section 2 above include proton wires that may be part of a network that includes water and protein. For example, bacteriorhodopsin proton transport requires both water and a side chain flip [83,84]. Trofimov and coworkers have shown the existence of water pools in the temperature sensing TRPV1 channel [85]. A central question for the role for water wires in channels concerns the hydrophobic barriers that are often cited as interrupting the passage of protons through a channel; such a barrier, for example, seems to be an argument against a proton-gating mechanism in the VSD of a K_V_1.2 channel. However, an interesting modeling effort by Kratochvil and colleagues [86] shows that water fluctuations can bridge such gaps; the bridge may be short lived, but it would be adequate to allow a few protons, enough for the gating current, to pass through. A simulation study [47] found a water cluster in the center of the H_v_1 pore but not in the VSD of a potassium channel. However, even putting aside questions about MD studies, fluctuations between the upper and lower water pools may make it possible to have transient proton passage. Considering that only about three protons would have to pass in perhaps tens of microseconds, this would not be a large enough current to be blocked (in macroscopic terms, it would correspond to a transient current on the order of tens of fA). In addition to possible fluctuations, side chain rotations may be required in some cases. The influenza channel just discussed seems to require a histidine side chain to move and to ionize to different charge states to allow proton passage [87,88]. Water wires are not limited to channels, but may appear wherever proton transfer along a protein is required for function; a paper by Shinobu and Agmon shows this in the green fluorescent protein [89]. A combination of fluctuations in water surrounding a protein, coupled to the fluctuations of the protein, especially side chains, has been calculated for human carbonic anhydrase II [90,91]. Proton transport may also be coupled to an anion, as in the Cl^−^/H^+^ exchanger [92]. In cyt c, the proton transport is coupled to electron transport [93], and specific side chains again act as a sort of gate, coupled to fluctuations. These examples suffice to show how there are proton transport wires in multiple proteins, and that these are coupled through water and the side chains of proteins. The proposed proton chain in the VSD of K_V_1.2 is not unusual and behaves like normal proton transfer chains; it is limited to only about three protons because no more are available. The existence of a proton current with a single mutation is just what would be expected when protons are available. Fluctuations near a protein surface, especially the hydrophobic sections, are difficult to sample, but a method has been proposed for sampling that is more efficient [94]. Even defining a hydrophobic section requires care, as the water lone pair electrons on the oxygen can interact favorably with aromatic π electrons [95], and so the orientation of water at the surface and the orientation of aromatic side chains should be taken into account in deciding what is hydrophobic. More generally, water networks may span a protein, including one with hydrophobic sections; certain hydrophobic interactions are key to some protein functions [96]. Networks percolate across a surface, for example of a protein, and it has been suggested that the biological function depends on a percolation phase transition [97]. Ions can also interact with each other, as mediated by water [98], and we will come to that in the next section.
*(4)*  *Ion hydration in confined spaces, like channels:* Ions must pass through the gate of the channel and then through the pore. The ion pathway in the channel can be considered in three major sections (this is not the only possible choice of ways to consider the path, but it is the simplest and most direct). (i) Start with selectivity: selectivity of the K_V_1.2, and other potassium channels for potassium over sodium, appears to largely depend on a selectivity filter (SF) that is conserved from viruses through both domains of protists and essentially all eukaryotes. The filter has a sequence of amino acids (T)TVGYG, where all but the first T are essentially absolutely conserved [99], and the first T nearly so. For channels for which the ion path is from the intracellular to the extracellular space (this includes delayed rectifier channels like K_V_1.2), this SF is the last section, with the ion leaving from the end of the filter to the extracellular space. There have been a myriad of papers, mostly theoretical, on the passage of the ion through the pore. There are four positions in the filter that can be occupied by the ion or else by water. Determining the number of spaces occupied by ions, as opposed to those occupied by water, has been a struggle for years. A potassium ion in bulk water will have, most of the time, around six water molecules in its hydration shell. The energy of the water interaction decreases as the number of water molecules bound to the ion increases; this is most easily seen for the gas phase, where it was measured by Kebarle and coworkers half a century ago (Table 1 [100]).

It may seem odd to include gas phase thermodynamic quantities here. However, while the gas phase numbers are not going to equal the liquid phase numbers, they do suggest the order of strength of adding hydration. Not surprisingly the first hydration is the strongest, and sodium is stronger than potassium—with the anions, we can see that the hydration is weaker. Kebarle [100] also gave Gibbs energy differences for the cations but not the anions. The Gibbs energy differences essentially tracked the enthalpy differences. The gas phase hydration strength is more or less in line with what we expect from the size and kosmotropic/chaotropic series. These numbers are unlikely to be relevant for bulk water, where the exchange rate of tens of picoseconds makes the fluctuations too fast to be of any biological interest. However, the state of hydration in the gate of an ion channel would be much slower, and the number of water molecules hydrating an ion would be more like two for K^+^ and three for Na^+^ in the confined space of a channel pore. The gas phase differences in enthalpy for adding a water molecule are likely to be similar to what they would be at a channel gate, and it is therefore worth considering for a qualitative understanding of the order in which hydration may take place in a pore, in which it is necessary to strip off some of the hydrating water. Inasmuch as the gain in energy for adding a third water to a K(H_2_O)_2_^+^ is about the same as the energy to add a first water to chloride, it would be interesting to know the interaction energy in the pore of K(H_2_O)_2_^+^ with Cl^−^—do we obtain ion association? In the 1950s and 1960s, Raymond Fuoss and coworkers measured such quantities in bulk solution, but we have not found measurements of these in constrained spaces. Another point that would be interesting would be to substitute Br^−^ for Cl^−^ in some physiological experiments, but this experiment also does not seem to have been conducted.

However, in the SF, there would be space for just two water molecules per ion. We will see that potassium near the gate must also get down to two water molecules. There are two relevant experimental papers. One of the chief questions has been the number of water molecules per ion in the selectivity filter. The way to measure this is via the streaming current, which gives the ratio of K^+^/H_2_O directly. This measurement has been carried out in one paper, and, as with everything having to do with channels, it is complicated (Iwamoto and Oiki [101]). When the intracellular side of the channel has a high enough concentration of K^+^, the ratio is 1:1, so presumably the occupancy of the selectivity filter alternates between K^+^ and water. The four states are occupied as K/W/K/W. However, if the potassium concentration is dilute, the ratio is 2.2. This is quite different to what is found for the carrier (carriers complex with the ion and carry it across the membrane) valinomycin [102]. The second paper is also relevant to selectivity. If a Na^+^ gets into a channel, it largely blocks the channel. While this basic phenomenon has been known for half a century, it was measured quantitatively on a KcsA channel [103], a bacterial potassium channel that has a smaller central cavity, with fewer water molecules, than K_V_1.2. It is consistent, however, with the Iwamoto and Oiki paper on streaming currents in finding different binding mechanisms for the concentrated and dilute solutions of potassium. (ii) Ion hydration in bulk or, for that matter, in vacuum is fairly well understood. The binding energy and free energy of water molecules was determined in vacuum (Table 1), and it was found that each water is less strongly bound than the previous one. In solution, it appears that both the K^+^ and Na^+^ ions usually have six water molecules, although this fluctuates, and partners exchange on a tens of picoseconds time scale. Potassium is structure breaking (chaotropic), while sodium is structure making (kosmotropic). In other words, Na^+^ holds its hydrating molecules more tightly than K^+^. This has consequences as the ion approaches the gate. It appears that this has not been carefully examined in the literature. However, if we assume that the gate diameter in the closed state is not much different from that in the open state (appropriate for the proton gating model, but not the standard models), then the changes in hydration between the two types of ion are important. Probably, they should be important even for the standard models, although this seems not to have received a great deal of attention.

*A role for D_2_O?:* If the binding of a water molecule to anything, ion or protein, is relevant at all, one should expect that D_2_O would matter. Several experiments have shown that it does. Because no structural information is needed to determine the main electrophysiological properties of gating, the first tests were quite simple. Using D_2_O was shown by Schauf and coworkers to slow gating in a potassium channel by inhibiting the fast phase of gating [104]. Earlier still, the same group showed gating in a sodium channel was slowed by D_2_O [105,106,107]. Furthermore, they were able to separate phases of gating by finding different effects of D_2_O on different phases of gating. Starkus and Rayner and coworkers found at least two stages in gating that were affected by D_2_O substitution; this included the finding by Alicata et al. that the last step in gating a sodium channel was slowed by D_2_O [108,109,110]. Potassium channels also have solute inaccessible changes during gating [111]. The interpretation of the D_2_O results is actually not entirely simple. However, it is clear that these effects exist. Because of the importance of water in the transport of protons and the fact that several steps, from VSD to linker to gate, are involved, the water/proton gating model can make at least two strong predictions with respect to these effects: first, they must exist; second, they must show different stages, as protons must move through three stages, gate, linker, VSD. Both of these predictions are fulfilled. Unfortunately, they are not sufficiently specific as to allow strong conclusions to be drawn. In the consensus model, it is difficult to understand why isotopic substitution in the solvent should matter, if the solvent did not take part in gating. The existence of D_2_O effects is necessary, but not sufficient, for the proton gating model. This necessary condition is clearly fulfilled. For the alternative, it is difficult to explain why the solvent is of importance, but possibly a version can be found in which the solvent plays a role. The consensus models do not make clear how the solvent could play a role at all. Conceivably, even on a standard model, the hydration of the ion might matter, although how this might matter for different steps in gating, as was shown by experiments, is not obvious. On the standard model, one might speculate that the tighter binding to D_2_O might diminish current, but slowing gating seems harder to account for. So far, we have said very little about inactivation, especially slow inactivation. However, there is evidence for the existence of a cluster of structural water molecules behind the selectivity filter that slows K^+^ ion rehydration [112,113], structural water appears to play a role throughout the channel. D_2_O is also involved in slowing C-type inactivation, in a manner involving hydration of the ion.

*Fluometry:* Evidence from fluometry has been interpreted in terms of the standard model, but in the end, the conclusion has been that more work is needed. Two kinds of effects can be measured: solvatochromic shifts in spectra brought about principally by changes in local electric field, and FRET-type quenching experiments that should depend on the distance between the absorbing and the fluorescent moieties. The most important solvatochromic result of all is that of Asamoah et al. [43] that showed that the voltage dropped almost all at one arginine in the VSD, which is consistent with our calculation of the R-E-Y proton source. The interpretation of FRET (or the improved LRET variant) studies show that something changes at the gate and sometimes in the VSD. There is considerable additional literature on fluometry on ion channels [114,115,116,117,118]. Even for FRET type studies, however, the interpretation is complex, as the rotation of either the absorber or the fluorescent moiety, which are generally of the order of size of the effect that is supposed to be measured, could produce the entire effect. Without reviewing the entire literature on this subject, we can state that it is not necessary to agree that this requires interpretation in terms of the standard model. The latter requires a large mechanical displacement of the S4 segment. Side chain rotations and field changes occasioned by the change in protonation state, for example, could also produce the observed effects.

*Simulations*: The standard model has been ostensibly supported by a huge amount of the literature on simulations. We have cited a couple of simulation studies, especially with reference to the Hv1 channel. We mostly omitted discussions of these, as we had criticized problems with these studies in an earlier work, most recently in some detail in an article in Symmetry [119]. The difficulty with classical simulations, in practically all cases, starts with the inability to observe the motion predicted by standard models. The response has been to use, as the reviewer observes, an order of magnitude too large a field, with the implied (almost never stated) assumption that the field effects are linear and using the excuse that the time scale is just being speeded up. The implied assumption of linearity is incorrect; at above about 10^7^ Vm^−1^, non-linearities begin to appear, such as the second Wien effect. The Onsager paper [44] on this is still approximately valid, even though it assumed a homogeneous dielectric constant and bulk solution. The Asamoah [43] paper mentioned in regard to fluorometry showed that the field dropped almost entirely across one of the arginines. The huge field used in the simulations would produce unknown effects at the other arginines, effects that are very unlikely to exist in reality. In a sense, there is a partially compensating error, because the classical simulations cannot allow ionization (this would also prevent the simulations from finding a proton current if one exists). Salt bridges, however, can be torn apart by high fields in ways that realistic fields could not accomplish. The treatment of water is highly uncertain, as many simulations use TIP3P water which is not a good model, especially at high fields where polarization is important. For these reasons, we have not cited much of the classical molecular dynamics simulation literature.

*(5)*  *A few mutations:* There have been far more mutation experiments reported than could be reviewed here. Only a limited number are directly relevant. The entire standard model began with the finding that substituting cysteine for arginine made it possible to determine the side of the membrane from which the substituted residue was accessible. The interpretation ignored the size difference between the tiny cysteine side chains and the large arginine and the fact that water could penetrate the VSD, so that cysteine could ionize in situ without moving to the surface, as was necessary for the standard model. Given these assumptions, accessibility required S4 motion. If the penetration of water, and the size difference of cysteine and arginine side chains, are taken into account, the interpretation of accessibility in terms of motion ceases to be obvious. Here, we will be concerned with certain mutations of residues that are conserved and ionizable, so that they are possibly relevant to the presence and transmission of protons. These include the finding of Lee et al. [120] that a glutamate in the S4–S5 linker is required for the channel to function. Lee and coworkers found that in addition to E327, a histidine, H418 (in the 3Lut numbering from the pdb), played a key role in the pH gating of the K_v_1.2 channel, both near the junction of the S4–S5 linker (i.e., the linker between the VSD and the gate) where the linker joins the pore below the gate. Results on the C-terminus of the K_v_1.2 channel [121] help to confirm this interpretation. Given their positions, it is easy to see how they must be part of the path through the linker for protons, as they enter the gate section. If protonatable residues were not present at the location where the linker joins the gate, it would be difficult to see how to have a proton path. The fact that these are absolutely necessary and well conserved suggests that there is in fact a proton path, as these residues have no special mechanical properties that would make it difficult to replace them with other residues of a similar size. Without these residues, the channel does not function. It is not obvious why in the standard models, in the absence of a proton path, these would be critical. Second, in a pair of papers from Swartz and coworkers [122,123], it was shown that a substitution of aspartate for proline (P→D mutation) made the channel constitutively open at all physiological voltages (other similar mutations produced a lesser effect in this direction). They concluded that the channel underwent some sort of transformation that opened the channel even without a gating current. On the model we are proposing, the aspartates (there are four, one per domain) absorbed up to four protons, neutralizing the gate, with the consequence that the channel was open—the protons no longer formed a barrier to the progress of the positively charged K^+^ ion. The pore diameter distances in the open channel are still about the same in the open channel X-ray structure when aspartate is present as when the original, highly conserved proline, is.

We have restricted this discussion to very few mutations that appear to require sharply different interpretations from the standard model than from the proton gating model. These we find are consistent with the proton model, not with the standard model.

*(6)*  If the hydration of ions and the activity of water are important, then it must be the case that osmotic effects are significant. If the osmotic strength on one side of the membrane increases, so that the activity of water that is accessible to that side of the membrane changes, several processes change. Osmotic effects are well known. For one thing, the osmotic pressure can do mechanical work, and there is a large class of channels that are mechanically sensitive; we have not discussed these here, although there is evidence that their gating amounts to breaking a water column in the pore [124,125,126,127] (this list is not at all comprehensive but just offers a few examples). There are a number of channels, of types other than the type we are emphasizing here, on which similar experiments have been conducted [128]. Diaz-Franulic et al. combined osmotic pressure, streaming current, and viscosity experiments to suggest that water displacement during gating of the K_V_1.2 channel was comparable to that in slow inactivation, as well as to suggest that water displacement was important in both [129].
*(7)*  *Aquaporin channels—water channels:* There is another class of channels that can deal with osmotic stress, the aquaporin (aqp) channels. These transmit water in response to osmotic gradients. The 2003 Nobel Prize was shared (with Roderick MacKinnon) by Peter Agre, who discovered them. Hundreds of specific aqp channels are known in multiple families. The aquaporins are close to being ubiquitous in plants and are found in all domains of life. They are critical in the lens of the eye [130]. These channels have an interesting property in that they transmit water without transmitting protons, a trick that has led to a fair amount of puzzlement. One guess is that these channels have proton-transmitting water chains that double back, so that protons cannot be transmitted to the other side of the membrane, but return to the side from which they began. Other suggestions for the mechanism of action of these channels involve the electric field and free energy of the ions [131]. A more recent work has made different suggestions [132]. Overall, this class of channels has been the subject of a huge number of studies concerning their location as well as their mechanism of transmitting water while not allowing the transport of H^+^ [130,131,133,134,135,136]. There are several suggestions for detailed mechanisms that involve lipids and phosphorylation, among other factors. The existence of these channels is to be expected, given the central role that water, and the activity of water, plays in maintaining the conditions that allow cells to function.

*Some recent calculations on the path of the potassium ion near the gate, and the coupling to the S4–S5 linker.* We have carried out quantum calculations on the VSD, the S4–S5 linker, and somewhat incomplete but already informative calculations on the region near the gate. Certain mutations in the gate region, especially the proline to aspartate mutation we discussed above in the highly conserved PVPV sequence at the gate, produce a channel essentially constitutively open [122,123,137] at physiological potentials. These are qualitatively relatively easily understood on the proton model, but not on the mechanical model. There is no reason this P→D mutation should produce a wider gate opening, but the aspartate can take up protons with its carboxyl. This mutation also produces a larger current than the wild type, and the hydration of the ions near the gate is likely to be critical; we discuss hydration below. We will not review all the linker mutations that have been reported but only note that these have led to several contradictory models of the VSD–gate coupling, none of which appear to be close to accounting for all the data. The proton model that we propose does not contradict any of the evidence and is at least plausible for essentially all that has been reported—hydrogen ion transmission can occur under fairly general conditions.

Taken together, the evidence suggests that the standard model of voltage gating is not proven by the available evidence. It is also not completely defined, as the connection of the S4 motion to the gate requires the S4–S5 linker to make some specific conformational changes or cause the gate to do so. There have been some partial hypotheses, for example, by Blunck and coworkers, as to how the S4–S5 linker connects the S4 motion to the gate, including the suggestion that 3–4 Ả motion is sufficient [138,139]. These results are based on the effects of certain mutations, together with molecular dynamics, and do not appear to account for the effects of water. These include a role for the hinge motion around a glycine residue [140], an apparent crevice into which a residue from the linker fits (albeit in a related channel) [141], and modification of gating by PIP2 of the interaction with the T1 moiety, among other problems. A number of other hypotheses for the role of the S4–S5 linker have been made that are not compatible with each other. Related phenomena have been suggested for other channels [142,143]. It seems safe to say that the S4–S5 linker is critical to the gating of many channels, but that the exact mechanism is not yet a settled question. We have earlier shown how the VSD could produce a proton cascade [25], that the linker acts to conduct protons to the gate, and have found two paths that cross in K_v_1.2 by which this can happen [144]. These paths account for the role of water and that of the T1 moiety, which is known to be involved in gating [145]. It remains to show how the protons, once they reach the gate, close it. However, our model assumes that without protons, the gate allows K^+^ ions to enter. The standard model has always assumed an absence of mobile protons, at least as contributors to the gating current, so that it does not differ greatly from our model in the open state itself. Both models use the structure determined via X-ray crystallography; this has always been assumed to be the open state, as it has no voltage across the membrane. The major efforts and multiple hypotheses have been directed at finding the closed state; in the standard models, these hypotheses essentially always include a displaced S4 segment of the VSD. The models differ in the nature of the gating current, the function of the linker, and the nature of the gate in the closed state. In other words, all the dynamics are different, as well as the final result that produces the closed state. Here, we have considered the possibility that protons and water are central to this process. We have incomplete calculations at the gate itself that further support the proton gating model to the extent that they have been completed. The overall mechanism requires considering the properties of water in the neighborhood of a protein, the transmission of protons, and especially the hydration of the ions themselves. It also means looking at possible ion–ion interactions, including those with the most common anions in the cell, chlorides. Other relevant data include osmotic effects, isotope (D_2_O) effects—we have already mentioned something about D_2_O effects—as well as hydrogen bonding in the gate, and pH effects. We have given somewhat less weight to molecular dynamics results as these seem questionable for this system [117]. Only then can we summarize what appears, at this point, to be the preponderance of evidence.

**Figure 3:**
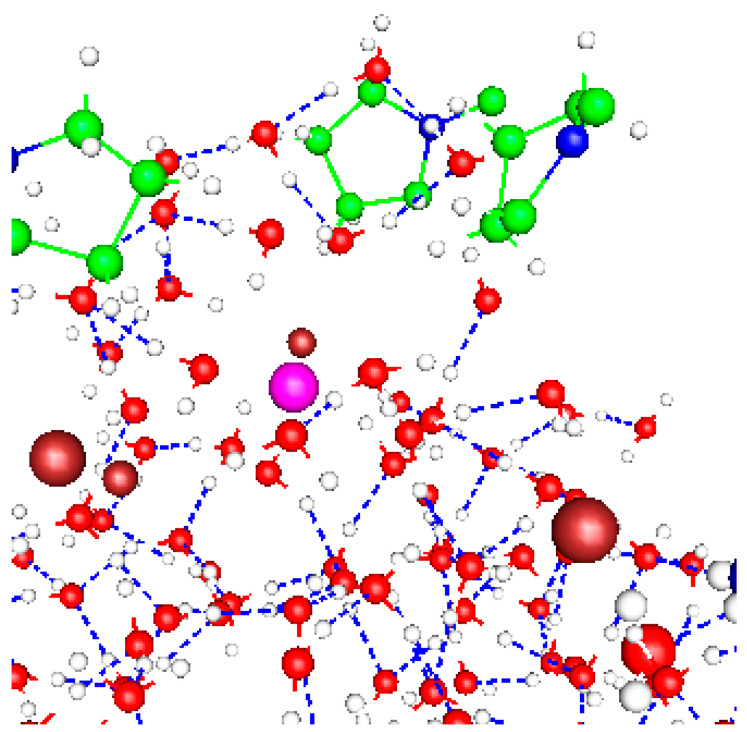
A section from the 1322 atom optimization, with the K^+^ near the level of the PVPV sequence; the calculation includes 10 added H^+^ and 3 Cl^−^. The K^+^ ion is purple, near the center. Two Cl^−^ ions (red-brown) are on the left and right, and rings at the top are proline (green, with one blue nitrogen). The red and white molecules are water (red, oxygen, white, hydrogen; other hydrogens, on the prolines, are also white) with hydrogen bonds showing. The K^+^ is still mostly hydrated, but a third Cl^−^ is in the background. K^+^ will have only two water molecules as first shell hydration as it passes the prolines. The hydrogen bond network is shown in blue dashed lines. Because so many water molecules are present, it is hard to see the Eigen and Zundel ions, which in any case are linked here.

The hydration of the potassium ion is an issue that has been discussed by a number of authors over the past several decades, as it is a question of obvious importance in a number of contexts [21,145,146]. The diameter of the hydrated ion is different from that of the bare ion, and a partially hydrated ion can rotate to provide a greater or narrower profile to fit through the pore at the gate. Figure 3 shows a section near a K^+^ ion cropped from a 1322 atom optimization, showing the local solvation near the K^+^ with 2 Cl^−^ ions nearby.

**Figure 4:**
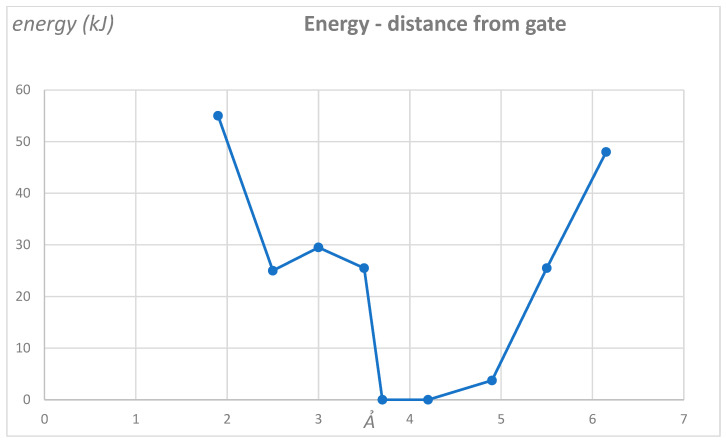
The energy of a system with a single potassium ion at nine positions approaching the PVPV gate of K_v_1.2 starting from the open, 3Lut, structure, including those amino acids near the gate, with some backbone protein atoms frozen to compensate for the absence of the remainder of the protein that would otherwise anchor the amino acids that are included. Ordinate energy in kJ, and abscissa approximate distance from the near “plane” formed by the PVPV sequence at the gate (Ả). The protein structure has ten protons added, but the positions of the amino acids at the gate hardly change at all from the open structure. The position of the “plane” of the PVPV sequence is not well defined (±0.5 Ả), but the distances between the potassium positions (dots) are correct to 0.01 Ả. There is a deep energy well, >20 k_B_T, and possibly much more (having made the point, we did not continue the calculations beyond these positions), and a few Angstroms from the gate when hydrogen ions are present. The net charge on the system as shown is +3, with four Cl^−^ ions in the system. Altogether, the system contained 1322 atoms, including 133 water molecules, the four Cl^−^, the K^+^ ion, 10 H^+^, and 908 atoms from protein, which included some charge among the amino acids. While this cannot be taken as definitive proof of the channel closure, it clearly shows that this is the most reasonable interpretation.

We have already shown how the protons could be generated in the VSD and cited the analogy with the H_v_1 channels to reinforce the point that protons could be generated and conducted through the VSD [25]. Figure 4 shows the energy of the system near the gate as a K^+^ ion approaches the gate, with ten protons having been added to the open structure of the channel near the gate. These calculations (see Appendix A for the method) provide evidence that protons can block the gate. Coupled with the linker calculations [10], we have a nearly complete gating model. At this point, confirmation must await experimental determination of S4 motion or lack of motion. What does appear certain is that water and protons are a significant part of whatever mechanism is found.

Figure 4 shows that the open *structure* of the gate can still block a K^+^ ion when there are ten protons present. The open structure itself does not offer a generous space for the ion to pass, as the distance between diagonally opposed proline CH_2_ groups is only about 12 Ả, so the ion can go through with one water ahead and one behind, but it would be very difficult to pass with a third water. To some extent, this may help with selectivity against Na^+^, as Na^+^ would require three water molecules or otherwise a lot of energy to remove the third water (here, Table 1 may be relevant—even though the channel is not in the gas phase, the water molecules are sufficiently isolated so that the energy differences are very likely comparable. Unlike bulk water, there is only one contact between each of the two hydrating molecules and the next water). Because we have difficulty defining the actual open state (it might, for example, have up to perhaps four protons) we did not attempt to do a comparable calculation of the open state. However, since the open state is experimentally open, this is not an issue—the question was whether ten protons could close the gate, and the calculation shows that it does. The hydration of the ions makes a difference in how the opening works. One further comment with regard to the P→D mutation that produces a constitutively open channel: the aspartate, if it were to stretch directly toward the ion path, would leave an appreciably *smaller* opening than the prolines, almost certainly closing the gate, if the gate were mechanical. As it is, we have to depend on the aspartate being more flexible, so that it can allow the ion to pass by rotating slightly. Whether the aspartate charge neutralizes protons, or pulls in K^+^, the combination of flexibility and charge makes the channel constitutively open.

*A comment on a side issue—plausible but speculative, and important if true:* There appears to be little evidence concerning the evolution of voltage-gated channels, with their elaborate arrangement of voltage-sensing domains, linkers, and gate. Generally, one expects to see simpler forms in evolution prior to a more complex structure. In fact, if gating does depend on protons, there are such forms that one can point to: observe the similarity of the H_v_1 channel to the VSD, and the fact that the H_v_1 channel is normally a dimer.

If two of these dimers combined with a channel like the bacterial KcsA, which is gated with a drop in pH presumably by protons but also has some voltage sensitivity, one obtains something like a K_v_ channel, but only if in fact the K_V_ channel is proton gated. This proposed evolutionary history could not apply to the standard model, as that depends on a mechanical linkage that must be complete to function at all, as several pieces must all fit precisely together. The standard model seems to give no obvious path to K_v_ evolution from any simpler form, as far as we can see, nor are we aware of any evolutionary paths having been proposed in the literature. Obviously, there are differences between a system with two H_v_1 + KcsA channels and a K_v_ channel; for example, the KcsA channel opens with added protons, while the K_v_ channel closes with added protons. However, having an ion path that is determined by a reversal of the effect of proton positions does not require a major evolutionary step. The linker would have to evolve separately, but considering that there are many ways of creating a proton path, this should not be too difficult. In a mechanical model, the pieces must immediately fit mechanically correctly starting from some initial form of the channel, which is, on the face of it, far more difficult. In the absence of direct evidence, this can only be a suggestion. However, channel evolution may be a path that is worth investigating, especially as there seems to be less work on it than one might expect.

## 3. Conclusions

**Figure 5:**
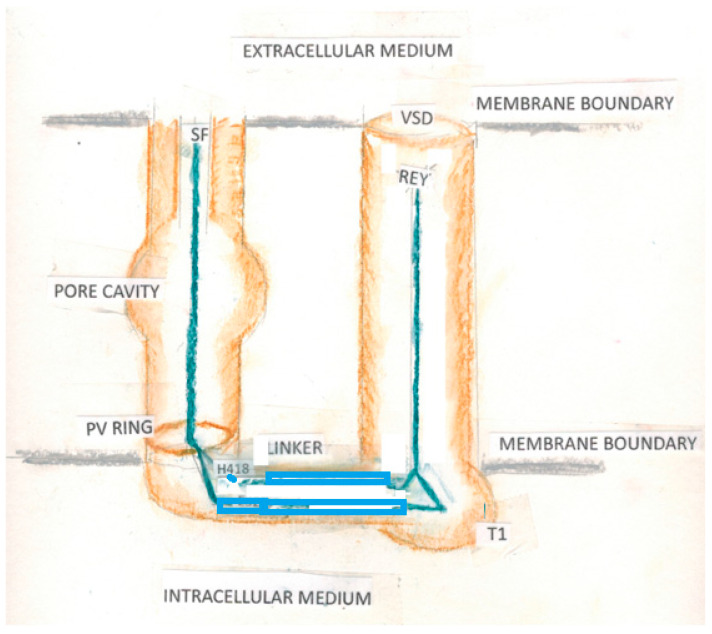
Summary cartoon of our proposed model: VSD = voltage sensing domain; REY, the arginine-glutamate-tyrosine triad that has the main voltage drop, and that produces the initial proton for the cascade; blue curves = possible proton paths; there are two that wiggle along the protein in the linker, one of which goes through the intracellular T1 moiety. E327, a key amino acid, would be behind H418 (E327 is not shown) H418 is a histidine where the paths come together; PV ring-proline, valine ring that is the narrowest part of the gate in K_v_1.2; above this is the pore cavity and the selectivity filter, not discussed here but included so that the complete orientation of the channel is clear. Orange clouds indicate the approximate location of protein boundaries, including the two intracellular linker chains, and the membrane boundaries are gray.

Cells must communicate with their surroundings. In the process, they may produce electrical pulses, as nerve cells do. They must maintain the activity of ions and water to maintain internal homeostasis. For this, there are a large variety of ion channels, as well as aqp channels for water. There are proton channels, as the pH must be maintained as well. Given the importance of these functions, it is not surprising that a huge effort has gone into understanding the channels. For the potassium channels, with which we are principally concerned in this review, there is a standard model of the mechanism by which the channels gate; while there are several versions that differ in detail, all versions require major conformational changes, especially for the S4 segment of the VSD. However, there are many phenomena that are difficult to explain using any version of the standard model. In particular, although water plays a role in some versions of the standard model, and sometimes even pH sensitivity, it seems difficult to find a coherent role for water in the standard models or for hydrogen ions. Therefore, we have proposed that the actual gating current consists of protons and have shown via quantum calculations that these give a consistent explanation for the gating of the K_V_1.2 channel. The extent of applicability to other channels is not discussed here, but is likely to be important. Figure 5 summarizes the model as it stands as of this writing; details are being filled in, and have already been filled in for the VSD and the linker, as in the references cited earlier. Calculations are still in progress on the gate, but have already shown that the gate provides a barrier to a K^+^ ion as it approaches the gate when ten protons are present. These calculations are also showing how the water and ions interact near the gate.

## Figures and Tables

**Table 1:** Enthalpy of adding hydration water to ions *.

N(H_2_O) **	H^+^	Na^+^	K^+^	Cl^−^	Br^−^
0→1	**	100.8	75.2	55.0	52.9
1→2	151	83.2	67.6	53.5	51.6
2→3	93.5	66.2	55.4	49.1	48.2
3→4	71.4	56	49.6	46.6	45.7
4→5	64.3	51.7	44.9		
5→6	54.6	44.9	42.0		

* Added waters: the ΔH (kJ) from adding one water in the gas phase to the ion with the previous number of water molecules, e.g., to add one water to Na(H_2_O)_2_^+^ to make Na(H_2_O)_3_^+^ has ΔH = 66.2 kJ/mol. ** Adding a single proton to a water molecule is not a meaningful comparison—it would be about 475 kJ, but this should not be compared to association of an ion that has electrons with water.

## Data Availability

Except for Figure 4, there is no new data; this is a review article. Data for Figure 4 are contained within the article.

## Appendix A

METHODS: Optimizations leading to Figure 4 used DFT optimization, with the NWChem program, with the following DFT and basis sets (calculations at Pacific Northwest National Laboratory, PNNL, using the Tahoma cluster): the calculations were conducted at the following level [147,148]:

basis “ao basis” spherical

* library def2-svp

end

basis “cd basis” spherical

* library “weigend coulomb fitting”

end

adft

xc xpbe96 cpbe96

disp vdw 4

tolerances

grid fine

convergence energy 1d-7
